# Development of a new high sensitivity mechanical switch for augmentative and alternative communication access in people with amyotrophic lateral sclerosis

**DOI:** 10.1186/s12984-019-0626-5

**Published:** 2019-11-29

**Authors:** M. Caligari, M. Godi, M. Giardini, R. Colombo

**Affiliations:** 1 Istituti Clinici Scientifici Maugeri Spa SB (IRCCS), Institute of Pavia, 27100 Pavia (PV), Italy; 2 Istituti Clinici Scientifici Maugeri Spa SB (IRCCS), Institute of Veruno, 28013 Gattico-Veruno (NO), Italy

**Keywords:** Augmentative alternative communication, Assistive technology, Amyotrophic lateral sclerosis, Rehabilitation, Neurodegenerative diseases, Motor neuron disease

## Abstract

**Background:**

People with Amyotrophic Lateral Sclerosis (PwALS) in the advanced phase are critically affected by an almost total loss of mobility and severe communication problems. Scanning access based on the patient’s interaction with a sensor (or switch) that intercepts even a weak body movement is a valid communication aid. However, its use becomes limited with the progressive decline of residual movements. To overcome this problem, we designed a new sensor, the Lever Magnetic-spring Mechanical Switch (LeMMS), allowing repeated activation/release cycles requiring a very small activation force.

**Methods:**

The LeMMS was applied and validated in a group of 20 PwALS in an advanced stage of disease. All subjects were regular users of communication aids employing other sensors, but which they could no longer operate their sensors (different from LeMMS). Patients were assessed at baseline (t0) and after one (t1), 6 (t2) and 12 (t3) months. Assessment at t0 included administration of standardized clinical scales, the Click-Test-30 counting the maximum number of LeMMS activations in 30 s, and thumb/fingers strength assessment with the Kendall scale. The QUEST 2.0-Dev questionnaire was administered at t1. Some use-related information and the Click-Test-30 were collected at t1, t2 and t3.

**Results:**

After one training session, all patients could operate the LeMMS with minimal residual movement of one finger. At t1, they used it on average 5.45 h/day. The mean score of the QUEST 2.0-Dev was 4.63, suggesting strong satisfaction with the LeMMS. Regarding Click-Test-30 scores, no significant difference was found between t0 and t1, but performance at t2 and t3 declined significantly (*p* < 0.005 vs. t0). At t3, 9/20 patients were still able to use their communication aid.

**Conclusions:**

This new switch sensor can enable PwALS to use their communication aids for a prolonged time even in the advanced phase of disease. It is easy to use, reliable and cheap, thus representing an intermediate alternative to more sophisticated and costly devices.

## Background

People with Amyotrophic Lateral Sclerosis (PwALS), or Motor Neuron Disease, experience a progressive loss of voluntary muscle strength in all body districts [1]. This includes the respiratory and phonatory muscles, causing a reduction of voice quality, speech speed and volume, with consequent difficulties in communication [2]. As a result, PwALS in the advanced phase are critically affected not only by an almost total loss of mobility but also by severe communication problems [3, 4]. About 95% of them become unable to speak at some point prior to death [5]. To improve their communication abilities, a number of Augmentative Alternative Communication (AAC) devices have been developed [6], thanks to which caregivers can understand their needs and provide appropriate assistance. These devices also enable patients to communicate socially with family and friends (reported as a primary need by patients), as well as to write/recount their personal experiences.

Modern AAC technology usually consists of electronic communication aids, which allow the use of picture symbols, letters, and/or words and phrases to create messages. Concerning this technology, a relevant point to consider is the increased life expectancy for PwALS who opt for invasive ventilation and percutaneous endoscopic gastrostomy. In practice, the decision to use invasive support techniques extends the overall length of AAC use. AAC devices are classified based on the access method they use. In general, the access method that is most suitable in an earlier phase of the disease differs as the disease progresses, so that patients usually need to change the access method in accordance with their residual abilities.

Access methods include: a) touch screen devices such as notebook or tablet; b) virtual keyboards activated by a special switch [7]; c) eye-tracking technology [8, 9]; d) brain-computer interface (BCI) [10–12]. Touch screen devices are very useful in the early phase of the disease, particularly in patients with bulbar onset, when arm and hand/finger movements are still preserved. As the neurodegeneration progresses and residual movements diminish (e.g. finger flexion, head/foot movements), virtual keyboards activated by an external switch become the method most frequently used for communication [13]. This is a common alternative text entry approach that uses a binary switch to select a row/column scanning a matrix of characters, symbols or images (often referred to as a spelling grid). With a fixed spelling grid that appears on a computer screen, the user selects a target symbol by simply activating the external switch when the desired row and, then, the desired cell is highlighted. With further decline in residual movements, eye-tracking and BCI remain the only access methods available. Although in the last decade eye-tracking technology has improved in reliability and the cost has decreased, its diffusion outside the clinical setting is still limited [9]. In addition, BCI is at an early stage of development and application, it has high costs and, in general, cannot be applied for a long period or outside of the user’s home. As a result, its diffusion amongst PwALS is low [14].

Due to weakness of the residual movements, patients have great difficulty in activating many of the switches available on the market – the operation can take quite a few minutes and causes fatigue. In addition, with disease progression, persons with ALS may have difficulty moving against gravity or may be able to move in one direction only. Consequently, there is a rapid decline in the production of written text.

Alternatively, one might consider the use of capacitive switches requiring a very low activation force. Unfortunately, after the switch has been activated (e.g. by forefinger flexion) the lack of the antagonist muscles (in this case, finger extensors) makes it impossible to return the switch to the rest (“before click”) position. In other words, the patient can touch the capacitive switch surface but cannot retract the finger after its activation so releasing the contact. Based on our experience, other sensor-based switch types such as breath and eye-blink activated switches have similar problems and their function fails or they require continuous adjustments to adapt to the disease progression, i.e. to find the new set-up threshold suitable for the current residual movement [15].

The aim of this study was to present a new switch sensor designed to allow repeated activation and release cycles by requiring a very small activation force and providing a return force suitable to move the finger against gravity. We report here the theoretical mechanical design concept of the switch, its implementation and validation through the measurement of use and satisfaction in a group of PwALS. In addition, to allow prediction of the sensor’s limits of applicability during disease progression, a new metrics is presented, and its sensitivity evaluated. We hypothesized that the LeMMS would allow patients to more easily exploit their residual abilities and maintain the ability to use their CAA system for a longer time. Further, due to its ease of use, low cost and simple transposition into the home setting, we hypothesized that the LeMMS would be perceived by patients and their caregivers as improving their quality of life.

## Methods

### Mechanical design

**Fig. 1:**
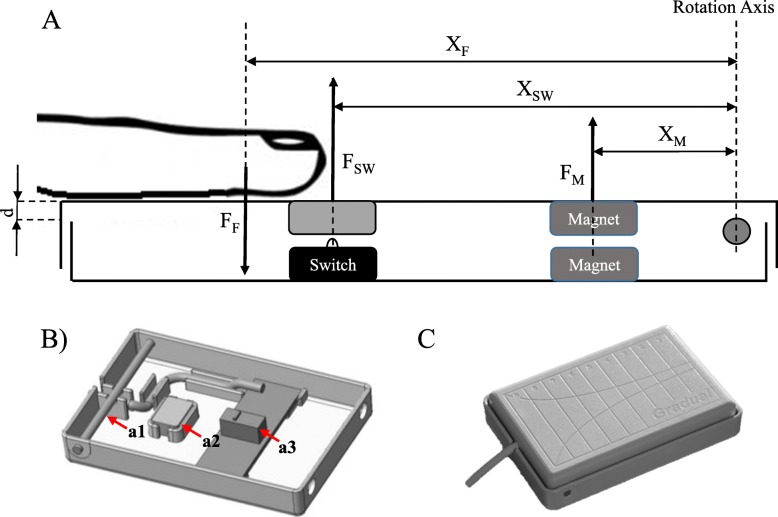
Diagram of LeMMS. **a** Schematic diagram of the LeMMS. *F*_*F*_ is the finger exerted force, *F*_*M*_ the repulsive force between magnets, *F*_*SW*_ the force exerted by the micro-switch spring and *X*_*F*_, *X*_*M*_, *X*_*SW*_ are the respective distances from the rotation axis. The vertical linear displacement d is the first approximation of the rotation angle. **b** LeMMS half-shell with brass pin (a1), magnet (a2) and micro-switch (a3). **c** Illustration of the LeMMS assembled

The Lever Magnetic-spring Mechanical Switch (LeMMS) was specifically designed to allow activation with a very low contraction force and to provide a return force suitable to move the finger against gravity **(**to not require a finger extension for switch deactivation**)**. It consists of two rectangular floating half-shells joined by a pin (with fulcrum function) at one end. A mechanical micro-switch (Huaweite Electronics Co. Ltd., mod. WK1–01), with a buckling-spring switch design, is activated by the slight rotation of one half-shell with respect to the other. A couple of magnets (Calamit© - Ferrite Cuboid Magnet, dimensions 9x5x5mm, adhesion 350 g. on 9x5mm^2^ surface) facing each other with the same polarity works as an additional spring providing the repulsive force required to maintain the switch open in resting conditions. Hence, the functioning of LeMMS is based on the equilibrium relationship of the moment of forces reported below. Figure 1 shows the forces involved during use of the device.

In order to activate the switch, the moment of the force exerted by the finger should be higher than the sum of the forces exerted by the return spring of the micro-switch and by the magnets.

The following formula represents the equilibrium relationship:

1 $$ {F}_F\ast {X}_F={F}_M\ast {X}_M+{F}_{SW}\ast {X}_{SW} $$

where:

*F*_*F*_ is the finger exerted force, *X*_*F*_ is the distance of *F*_*F*_ from the rotation axis, *F*_*M*_ is the repulsive force between magnets, *X*_*M*_ is the distance of *F*_*M*_ from the rotation axis, *F*_*SW*_ is the force exerted by the micro-switch spring and *X*_*SW*_ is the distance of *F*_*SW*_ from the rotation axis.

The finger-exerted force consists of two components, i.e.:

*F*_*F*_ = *F*_*WF*_ + *F*_*P*_ (where)

*F*_*WF*_ is the weight of the finger and *F*_*P*_ is the force actively exerted by the patient’s muscle contraction. Then from eq. (1) we obtain:

2 $$ {F}_P=\left[{F}_M\ast {X}_M+{F}_{SW}\ast {X}_{SW}-{F}_{WF}\ast {X}_F\right]\ast 1/{X}_F $$

If as a first approximation we consider *F*_*M*_, *X*_*M*_, *F*_*SW*_, and *X*_*SW*_ as constants that depend on the construction features of the device, it results that the force required to activate the device is a function of the finger position on its surface.

**Fig. 2:**
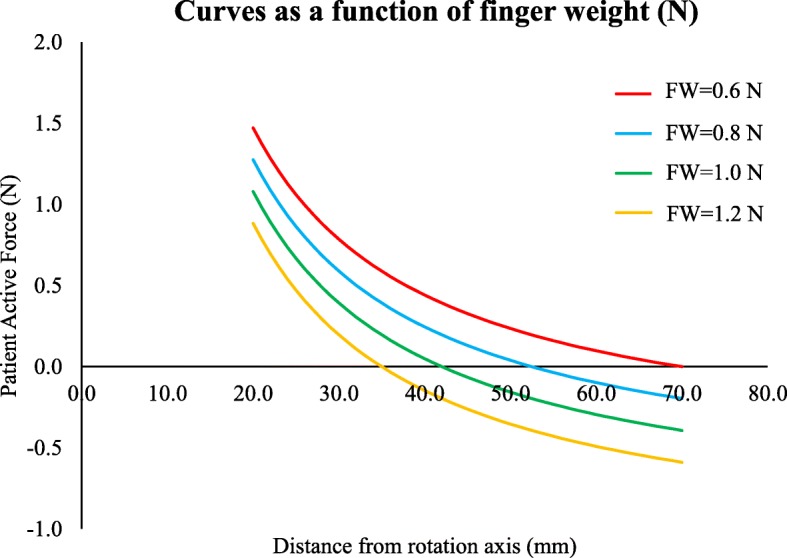
Patient’s active force as a function of the distance from the rotation axis. The curves represent the patient’s active force required to activate the LeMMS as a function of the distance from the rotation axis for different finger weights (respectively 0.6, 0.8, 1.0, 1.2 N). These values were synthetically selected from a realistic range of finger weights. When the patient’s active force is negative, then the switch remains always activated. In our case, to allow activation, the finger needs to be moved toward the rotation axis. In other words, independently of the finger weight, a small activation force of the LeMMS can be easily found by sliding the finger over the device surface

Looking at Fig. 2, one can see that a simple longitudinal sliding of the finger allows to find, in relation to the finger weight, the most appropriate distance from the fulcrum so that only a very small active force is required to activate the device (as little as a few grams).

Optimum force characteristics of a key require a steadily increasing force as it is depressed until contact is made. Immediately beyond the switch-contact point, the force is substantially changed so that users can “feel” when the key has been pressed [16].

Actually, the micro-switch spring force and the magnets’ repulsive force are not constants, but both depend on the rotation of the finger support plane. For very small rotation angles, the rotation can be approximated to a linear displacement (d) along the vertical axis (see Fig. 1).

The relationship between the key force and displacement of the micro-switch was assumed to be in accordance with the specifications reported in the ISO/DIS 9241 standard [17].

Further, the magnetic repulsive force depends on the distance squared for large displacements; in our case, the initial distance between the magnets is about 2.5 mm, which is reduced by 1 mm when pressed. Then, the repulsive force can be considered in a first approximation as linearly dependent on the displacement.

In order to provide a reliable model of the device we measured the actual characteristics of these force components using a simple experimental set-up including a micrometric surface gauge (NIIGATASEIKI VHK-15), and a load cell and bridge amplifier (HBM S2M, MVD 2420) [17, 18].

**Fig. 3:**
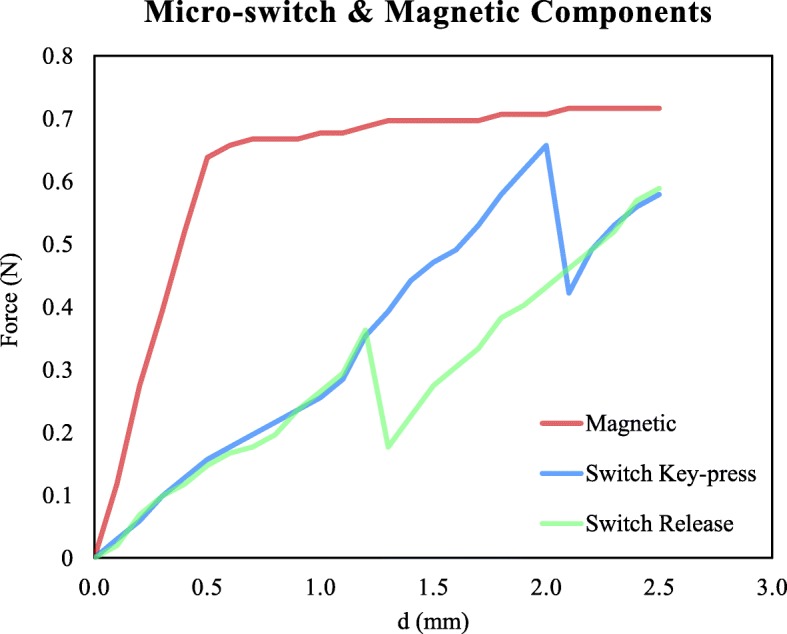
Displacement-force relationship of the micro-switch and magnetic components measured independently in a sample device. The red line represents the magnetic repulsive force component, the blue line is the switch force component for the key press condition, and the green line is the switch force component for the switch release condition. Forces are reported as a function of the displacement d (proportional to the rotation angle)

Figure 3 reports findings on the displacement-force relationship in a sample device. Repeated measurements on different component samples (same model #) revealed non-significant variability for the switch and magnets performance.

Both components can be modeled by a combination of linear elements and, then, eq. (2) is replaced by the following:

Key-press condition:

3 $$ {\displaystyle \begin{array}{c}{F}_p=\left[\left({F}_{M0}+d\ast {K}_M\right)\ast {X}_M+\left({F}_{SW0}+d\ast {K}_{SW}\right)\ast {X}_{SW}-{F}_{WF}\ast {X}_F\right]\ast 1/{X}_F\kern0.48em \mid for\;d>0.5,d\le 2.0\\ {}{F}_P=\left[\left({F}_{M0}+d\ast {K}_M\right)\ast {X}_M+\left({F}_{SW1}+d\ast {K}_{SW}\right)\ast {X}_{SW}-{F}_{WF}\ast {X}_F\right]\ast 1/{X}_F\kern0.48em \mid for\;d>2.0\end{array}} $$

Release condition:

4 $$ {\displaystyle \begin{array}{c}{F}_p=\left[\left({F}_{M0}+d\ast {K}_M\right)\ast {X}_M+\left({F}_{SW0}+d\ast {K}_{SW}\right)\ast {X}_{SW}-{F}_{WF}\ast {X}_F\right]\ast 1/{X}_F\kern0.48em \mid for\;d>0.5,d\le 1.1\\ {}{F}_p=\left[\left({F}_{M0}+d\ast {K}_M\right)\ast {X}_M+\left({F}_{SW1}+d\ast {K}_{SW}\right)\ast {X}_{SW}-{F}_{WF}\ast {X}_F\right]\ast 1/{X}_F\kern0.48em \mid for\;d>1.1\end{array}} $$

where:

*K*_*M*_ and *F*_*M*0_ are respectively the slope and intercept of the line modeling the linear magnetic repulsive force, and *K*_*SW*_, *F*_*SW*0_ and *F*_*SW*1_ are respectively the slope and the intercepts of the lines modeling the micro-switch spring forces during the key-press and release phases. It is worth noting that the linear model assumes the same slope for both phases and the presence of a hysteresis phenomenon.

All the elements of equations (3) and (4) except for *X*_*F*_, *d*, *F*_*P*_ can be considered as constants that depend on the construction features of the device.

### Validation procedure

#### Subjects

The switch was applied and validated in a group of 20 PwALS who contacted our Laboratory of Communication and Domotics at the Istituti Clinici Scientifici Maugeri IRCCS of Veruno, Italy, between 2013 and 2016, on account of communication problems or need for alternative access to computers, internet access and home automation activities. The patients (14 M/6 F; mean age 59.9 years) were in an advanced stage of disease with anarthria, as attested by a score of 0 (loss of useful speech) or 1 (speech combined with nonvocal communication) for item 1 (“speech”) on the Amyotrophic Lateral Sclerosis Functional Rating Scale-Revised (ALSFRS-R). All subjects included in the study were regular users of AAC with scanning access through a single switch sensor (different from the LeMMS) and/or used an emergency call system (Emergency Call Bell activation) during the day/night hours.

#### Assessment

All patients were assessed at baseline (t0) and after 1 (t1), 6 (t2) and 12 (t3) months of use of the LeMMS. Because of the expertise required for management and technology application with regard to PwALS, the study was not blinded.

#### Baseline evaluation (t0)

Demographic and clinical characteristics of all patients were collected at baseline, by two physiotherapists with expertise in AAC application. The same two physiotherapists gave instructions to the patients and caregivers on positioning of the LeMMS and assisted with optimization of the scanning access during the training session. The type of switch previously in use and the communication problems the patient was experiencing were noted in the patient’s medical record. In addition, the following evaluations and clinical scales were administered:

- The ALSFRS-R. This is a 12-item questionnaire with the score for each item ranging from 4 (normal function) to 0 (impossible to do). We calculated the total score (ALSFRS-R-tot), ranging from 48 (normal) to 0 (severe impairment) [19].
- In order to assess patient’s ability to activate the switch, the subjects were asked to click and release the LeMMS as many times as possible in 30 s. Using a chronometer, we recorded the number of clicks in the defined period and called this test: Click-Test-30.
- The strength of thumb and fingers of the hand used was assessed in each subject with a scale proposed by Kendall [20]. It is an 11-level Likert scale for muscle strength evaluation, where 0 indicates no muscle contraction and 10 normal strength. Each patient performed the test on the finger usually employed to activate the communication device or on the finger with best residual activation. The evaluation was carried out by the therapist involved in assessment by rating the requested maximal finger activation in isometric conditions.

#### One-month evaluation (t1)

After 1 month from t0, evaluations were performed by the caregivers in their home and transmitted by phone to a third physiotherapist.

The evaluation consisted of:

- The Quebec User Evaluation of Satisfaction with Assistive Technology (QUEST 2.0) questionnaire. It is a 12-item scale, which assesses users’ satisfaction with assistive technology devices. This scale investigates two dimensions: satisfaction with the device and satisfaction with the service. Item scores range from 1 (not satisfied at all) to 5 (very satisfied) [21]. Only the four items related to satisfaction with the device (QUEST 2.0-Dev.) were reported for this study.
- The Global Rating of Change scale (GRC) to detect self-perceived improvement in use of the emergency bell - it was evaluated in those patients who were equipped with this communication aid. The GRC is a single-item questionnaire, grading on a 15-point self-reported scale the perceived progress (or lack of progress) since the initial intervention [22]. The score ranges from − 7 (lack of progress) to + 7 (excellent progress).
- The Click-Test-30 to assess the patient’s ability to activate the switch. The subjects were asked to click and release the LeMMS as many times as possible in 30 s. In order to avoid bias, this measure was carried out by means of a dedicated software program that counted the number of LeMMS activations in the defined period. It is worth noting that both the therapist’s and the caregiver’s (at follow-up) interventions were limited only to activation of the test program and reading the measure.
- Some use-related information such as: daily usage time, purpose of use (scanning access or emergency bell), finger movements (flexion, abduction) used with LeMMS, set-up problems and possible side effects, i.e. discomfort, adverse contact reactions such as allergies, pressure sores, skin redness. In particular, the daily usage time was measured by means of a background running program included in the AAC system. The measured value and other information were reported by the main caregiver, based on specific questions during the follow-up call.

#### Follow-up at 6 (t2) and 12 months (t3)

After 6 and 12 months from the baseline evaluation, caregivers were contacted by phone to verify if the patients were still using the LeMMS. In addition, they were questioned about the Click-Test-30 value (see below) and if there were any communication system problems.

#### Intervention

The physiotherapists evaluated all patients enrolled in the study in order to identify the most appropriate finger movement required for activation of the switch, such as finger/s flexion or thumb abduction. Patients were provided with the LeMMS as a substitute for their previous switch, and it could be used to activate both the scanning access system and the emergency bell, as needed. In order to avoid learning problems with the new switch, patients maintained the same communication system and software previously adopted, in accordance with their needs and habits prior to this study. In other words, we did not oblige patients to use a different communication aid but changed only the type of activation device.

A training session lasting from 30 to 60 min depending on the patient’s residual ability was provided following the baseline evaluation. During this session, the patient and caregiver were instructed by the physiotherapist on how to find the optimal positioning of the LeMMS (replicating the same conditions a**s** at home). In order to fully simulate the usage scenario, the LeMMS was tested plugged in both to the bell and to the communication systems. In addition, scanning access speed was verified and if necessary optimized by the patient or caregiver under supervision by the therapist. Instructions about how to perform the Click-Test-30 were given to the caregiver, who was also invited to call our laboratory if there was any problem with the communication system (which could potentially indicate the need for an upgrade to more advanced technological solutions).

### Statistical analysis

All variables were expressed as mean ± standard deviation (SD). The number of activations of LeMMS during the Click-Test-30 was compared across the four time-points (t0, t1, t2, t3) by Friedman’s test. Paired comparisons were analyzed by the post hoc Wilcoxon test and the Bonferroni correction for multiple comparisons was applied (statistical significance being set at *p* < 0.008). Sensitivity and specificity of the Click-Test-30 as a predictor of the ability to use the LeMMS for scanning access was assessed by means of receiver operating characteristic (ROC) curve analysis [23]. The optimal cut off score was chosen as the point that jointly maximized sensitivity, specificity and ability in classified patients. In addition, the area under the ROC curve (AUC) was calculated as an indicator of the overall ability of the Click-Test-30 to predict an efficient use of the scanning access in our patients. The greater the AUC, the greater the Click-Test-30’s ability to distinguish patients able vs. unable to access the scanning system using the LeMMS. An AUC value > 0.8 was classified as an excellent discrimination power indicator. In order to assess the sensor’s limit of applicability, ROC analysis was conducted on Click-Test-30 and muscle strength values. The optimal cut-off point of ROC curves was estimated using the method proposed by Froud & Abel [24], which indicates the point on the ROC curve closest to the top-left corner of ROC space. Consequently, we selected the point in the ROC space that minimized the quantity q defined as: q = min {(1 – sens)2 + (1 – spec)2} [24]. The calculation was made using the STATA command “rocmic” followed by the “bootstrap” command in order to generate the confidence interval. Statistical analysis was performed using the STATA 13.0 (Stata Corp LLC, College Station, Texas, USA) software package.

## Results

The patients (*n* = 20) enrolled in the study had a mean ALSFRS-R score of 4.83 (SD 3.33). Sixteen were of the spinal subtype, 4 of bulbar subtype. Demographic characteristics of the patients are reported in Table 1.

**Table 1:** Demographic and clinical details of patients

Patient	Type	Gender	Age (yrs)	Disease duration (months)	Residual Movement	Strength of residual movement	ALSFRS-R	Previous Switch (failed)
1	Spinal	M	60	55	Ring flex	3	1	MicroLight
2	Spinal	M	62	84	Middle flex	4	3	Platform
3	Spinal	M	59	84	Thumb add	4	3	Pillow
4	Bulbar	F	61	24	Middle flex	3	9	Squarepad
5	Bulbar	F	73	33	Index flex	3	9	MicroLight
6	Bulbar	F	69	20	Thumb add	4	8	MicroLight
7	Bulbar	F	70	22	Index flex	3	7	Roundpad
8	Spinal	M	64	81	Middle flex	3	8	Roundpad
9	Spinal	M	42	38	Index flex	3	2	MicroLight
10	Spinal	M	44	30	Index flex	5	2	Pillow
11	Spinal	M	51	90	Middle flex	2	9	Plate
12	Spinal	M	84	31	Index flex	2	9	Plate
13	Spinal	M	67	29	Thumb add	3	1	Pillow
14	Spinal	M	42	68	Index flex	4	2	Pillow
15	Spinal	M	44	80	Index flex	4	2	Roundpad
16	Spinal	M	75	24	Thumb add	3	2	MicroLight
17	Spinal	F	50	22	Index flex	4	2	Pillow
18	Spinal	F	66	38	Middle flex	4	8	Squarepad
19	Spinal	M	50	60	Middle flex	6	17	Platform
20	Spinal	M	65	86	Middle flex	6	16	Platform
*Mean*			59.90	49.95	*Median*	3.50	5.00	
*SD*			11.97	26.27	*IQR*	3–4	2–9	

*IQR* Interquartile range, *flex* flexion, *add* adduction. Strength of residual movement was measured by the 11-level Kendall scale for thumb and fingers strength: score from 0 (= no muscle contraction) to 10 (= normal strength)

All patients reported that they were no longer able to use their scanning access system through the switch/sensors prescribed in a previous visit to our laboratory. In detail, 13 patients were using a mechanical switch (Platform®, Square Pad®, Round Pad®, Pillow®) requiring an activation force ranging from 1 to 2 N. Six of them considered the switch hard to press and fatiguing, while the other 7 no longer able to press it. Two patients were using a capacitive sensor. They could press (or, rather, touch) the switch at least once but could not release it because the antagonist muscles were not strong enough. Five patients used a type of sensor called Microlight®. This switch needs a very low activation force but, like the capacitive sensor, the return force it provides is too negligible to bring the finger back to the starting position after the activation click.

The communication system consisted of a scanning software (The Grid2®, CLICKER5® or ScanMouse). Fourteen out of 20 patients were also regular users of an emergency call bell, i.e. a call-for-help device usually activated via a specific facilitated switch similar to that used for communication. Switching between the communication and the emergency systems was provided by the caregiver for use during the night or resting periods.

After the training session carried out at the end of the baseline evaluation, all patients were able to use the LeMMS by virtue of the minimal residual movement of one finger: index finger flexion (*n* = 8), middle finger flexion (*n* = 7), thumb adduction (*n* = 4) and ring finger flexion (*n* = 1).

Table 1 reports the strength score evaluated for each subject at t0. On average, the group had very low strength (median value = 3.5).

The average training time, including that for explanations/training given to the caregiver, was approximately 45 min (SD 12.38) (Table 2). The Click-Test-30 at baseline showed that 12 subjects were able to activate and release the sensor > 20 times, 6 were able to do so 15–20 times, while 2 < 15 times. The table shows also that in the period between t0 and t1, patients used the LeMMS on average 5.45 h/day (SD 2.84). No side effects or set-up/technical problems were reported during the follow-up. Twelve patients used the LeMMS both for scanning communication and for emergency calls during the night; 6 patients used the LeMMS exclusively for scanning communication, while 2 patients were able to use it only for emergency calls. These 2 patients were the same ones who could activate and release the sensor less than 15 times at t0. The mean score of the QUEST 2.0-Dev was 4.63 (SD 0.26), suggesting high satisfaction with the LeMMS. For each item, the satisfaction score was greater than 4. The median score of GRC relative to the facilitation induced by the LeMMS in the use of the emergency call bell was very high, a score of + 6 out of a range of − 7 to + 7.

**Table 2:** Results of augmentative and alternative communication intervention

Patient	Training time (min)	Click Test at t0 (s)	Hours of use per day	Purpose	QUEST 2.0-Dev	GRC
1	60	23	8	Scanning+bell	4.88	6
2	45	25	8	Scanning+bell	4.75	5
3	45	26	8	Scanning+bell	4.63	6
4	60	24	2	Scanning	4.38	–
5	30	20	8	Scanning+bell	4.63	6
6	30	18	1	Scanning	4.50	–
7	60	19	4	Scanning+bell	4.63	7
8	45	23	6	Scanning+bell	4.75	6
9	30	21	7	Scanning+bell	4.88	7
10	45	32	7	Scanning+bell	5.00	6
11	60	11	7	Bell	4.25	6
12	45	13	7	Bell	4.88	6
13	60	18	1	Scanning+bell	4.00	5
14	30	25	8	Scanning+bell	4.75	5
15	30	27	8	Scanning+bell	4.25	5
16	45	18	1	Scanning+bell	4.63	6
17	60	19	2	Scanning	4.63	–
18	60	25	6	Scanning	4.75	–
19	30	65	8	Scanning	5.00	–
20	45	47	2	Scanning	4.50	–
*Mean*	45.75	24.95	5.45	*Median*	4.63	6
*SD*	12.38	12.02	2.84	*IQR*	4.50–4.82	5–6

*IQR* Interquartile range. GRC was administered to detect the self-perceived improvement in use of the emergency bell only

**Fig. 4:**
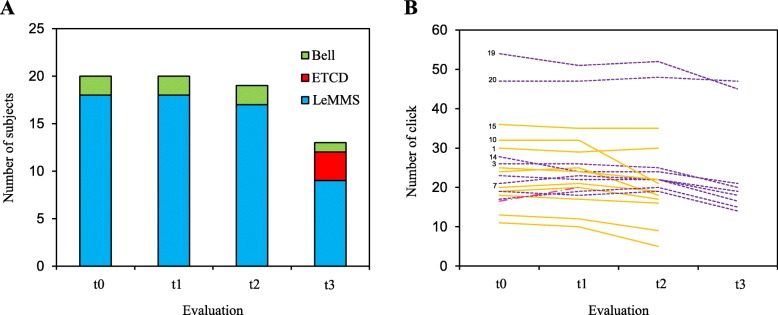
Usability of LeMMS compared to other modes of communication and PwALS’ performance on Click-Test-30 across the 12 months of the study. t0 = baseline evaluation; t1 = follow-up at 1 month; t2 = follow-up at 6 months; t3 = follow-up at 12 months. **a** Blue bars indicate PwALS able to use the LeMMS to communicate with scanning access. Green bars indicate PwALS able to use only an emergency Call Bell. The red bar indicates PwALS who switched from the scanning access to an ETCD. **b** Number of activations of the LeMMS obtained by each subject on the Click-Test-30 at the different time-points. Purple lines indicate PwALS who completed all follow-up assessments. The red line indicates the patient who dropped out between t1 and t2. Yellow lines indicate PwALS who dropped-out between t2 and t3

Figure 4a reports the number of subjects and their communication abilities with the LeMMS at baseline evaluation and at different follow-up times. At t2 (after 6 months), all subjects were still able to use the LeMMS except one who died at 5 months. At t3 (12 months), 9 patients (45%) were still able to use the LeMMS for scanning access and emergency purposes, while 7 had died (4 bulbar and 3 spinal) and 3 had switched to an Eye Tracking Communication Device (ETCD).

Figure 4b reports individual patient performances on the Click-Test-30 during the 12-month period of observation. The mean number of LeMMS activations in 30s decreased progressively across the four time-points (Friedman’s test, χ^2^ = 39.78, *p* < 0.0001). The mean number of activations was 25.2 ± 10.7 at t0, 24.9 ± 10.1 at t1, 22.3 ± 12.3 at t2 and 10.8 ± 14.8 at t3. Post-hoc analysis showed that the performances at t2 and t3 were significantly different from t0 (Wilcoxon post-hoc test, *p* = 0.003 and *p* = 0.0002, respectively for t2 and t3). No significant difference was found between t0 and t1 values (Wilcoxon post-hoc test, *p* = 0.51).

### Sensor’s limits of applicability

**Fig. 5:**
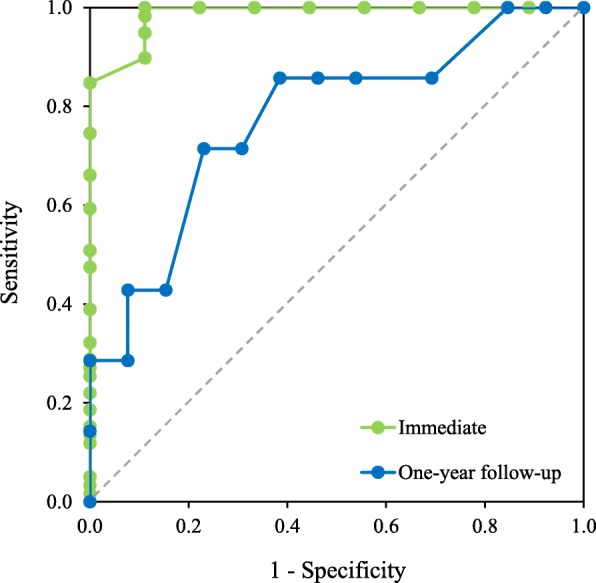
Receiver operating characteristic (ROC) curve of Click-Test-30. ROC curve of Click-Test-30 as a predictor of the ability to use LeMMS. Green line represents ROC curves of the Click-Test-30 considering the type of communicator used at t0 (immediate) and blue line represents ROC curves of ability at 1-year to use a communicator. The grey dotted line represents the random classifier and the AUC is equal to 0.5

We found a substantial correlation between muscle strength and the Click-Test-30, Rho spearman = 0.75 (95% C.I. = 0.47–0.90, *p* = 0.0001). The ROC curve analysis reported in Fig. 5 shows ROC curves of the Click-Test-30 considering the type of communicator used at t0 and at 1-year follow-up. The area under the ROC curves (95% C.I.) was 0.99 (95% C.I. = 0.96–1.00) and 0.77 (95% C.I. = 0.51–0.91), respectively, for current ability and ability at 1-year to use a communicator. The Click-Test-30 was able to differentiate patients who were able to use their scanning access system through the LeMMS from those who were not able to do so, with a cut off score of 16 (sensitivity, 1.00; specificity, 0.89; classified, 0.99; positive likelihood ratio, 9.00) at t0 and a cut off score of 24 (sensitivity, 0.71; specificity, 0.69; classified, 0.70; positive likelihood ratio, 2.32) at one-year follow-up.

ROC analysis was applied for prediction purposes also using the muscle strength variable. It could make a prediction with an AUC of 0.80 at t0 and 0.65 at t3. The AUC value at t0 was significantly lower (*p* < 0.0001) than that scored by the Click-Test-30.

## Discussion

The switch sensor specifically developed to allow repeated activation and release cycles for communication purposes in PwALS requires a very small activation force and provides a return force able to compensate the finger weight of each of the subjects we tested. The patients involved in this study could continue to use the same communication aids for a longer length of time simply by changing the activation device, so postponing the need for an upgrade to a more sophisticated technology. They could write text, surf the internet, manage the personal computer, and use the emergency call bell. Even though these patients were spending all their time in bed or in a wheelchair, they judged their access method as user-friendly and comfortable, as shown by the QUEST 2.0-Dev score. The high values of satisfaction reported by patients using a single switch sensor are comparable to those reported in the literature [25, 26].

Most patients enrolled in the study were able to use the single switch scanning for several months. In fact, only two out of 20 patients were unable to use their scanning communicator through the LeMMS, and this occurred after 6 months. In addition, the patients equipped with an emergency call bell were still able to use it thanks to the LeMMS. The failure in using the LeMMS for scanning communication appeared to be related only to the low activation/deactivation speed while for the activation of a call bell this parameter should not be relevant. In fact, based on our experience, when the speed of activation goes below a certain threshold the interaction is not feasible and the patient experiences frustration. Conversely, the activation of an emergency bell requiring a single activation remains a feasible task. The patients who were unable to use the scanning system were those who scored a low baseline value on the Click-Test-30 (see Table 2). The Click-Test-30 development was inspired by the “30s sit to stand test” which is widely used in neurorehabilitation to measure physical function and risk of falls [27]. Like other tests evaluating performance within a limited time interval, it should be able to provide a measurable value of the patient’s ability to use the LeMMS independently of his/her capacity to write using a scanning access device, so making it suitable for evaluation of activation of a simple device such as the emergency-bell.

The ROC analysis showed that patients who could press and release the LeMMS at least 16 times in 30 s were able to use the scanning system. This result is in line with Darvishi et al. who demonstrated that simple reaction time is correlated with BCI performance [28]. In other words, the simple reaction time and the Click-Test-30 (a measure analogous to simple reaction times, in our opinion) are compatible tests for predicting people able to operate their communication technology. It is plausible that subjects scoring high on the Click-Test-30 would have low reaction times. At t1, only two PwALS could activate the emergency bell and were recommended an ETCD system for their communication needs. The ROC analysis applied to predict the sensor’s limits of applicability showed that although the Click-test-30 and muscle strength are substantially correlated, the former is superior in predicting both PwALS who are able to use the LeMMS for AAC purposes, and those who will still be able to use it in the following 12 months.

Recent reports have discussed barriers and facilitators to the use of low and high-technology augmentative and alternative communication devices [14, 29, 30]. Some issues, not less important, are related to body functions. Here we discuss some key themes in the light of the advantages/disadvantages offered by the LeMMS in comparison to other solutions.

### Ease of use

Ease of use and of maintenance of the device is commonly reported by patients and caregivers to be a significant factor in enhancing the AAC user’s experience [29, 31]. Although more sophisticated technologies like ETCD and BCI may become fundamental when, for example, eye movements are the only volitional mode left for communicating, the value of a sensor switch is its easy application because the set-up is simple and does not require any calibration procedure. Thus, to prolong AAC usage as much as possible in the course of the progressive neurodegeneration, we retain that a solution such as the LeMMS involving a single weak residual movement should be considered as preferential.

In contrast to commercially available sensors that are not specifically designed for PwALS, the LeMMS offers the advantage of requiring a selectable small activation force combined with an easy design to control communication with a minimum residual movement. The very low activation force required is a feature shared with capacitive sensors, but the LeMMS allows selection of the scanned item even in the absence of finger extension ability. This feature is unique to the LeMMS – it is not possible with capacitive sensors. Of course, this aspect is crucial in the presence of finger extensor problems.

### Reliability

Cooper et al. described, in AAC usage, issues with the battery running out, devices being broken or not working, or devices losing the proper set-up within a short span of time [32]. A low reliability creates discomfort both to the patient, who temporarily loses the ability to communicate, and to the caregiver, who needs to repeatedly set-up the system. Of course, the switch sensor is the most critical part susceptible to mis-positioning. In accordance with Spataro et al., the long daily usage of the AAC measured in our study (on average 5.45 h/day) is typical of regular users and testifies to the excellent reliability of the LeMMS [33]. In addition, no specific problems pertaining to reliability of the system were reported by caregivers during the follow-up period.

### Availability of technical support

Family caregivers often describe difficulties with regard to the technical aspects of equipment requiring prompt intervention in the case of problems [31, 34]. This aspect is extremely important in the case of failure of the communication system and for this reason a secondary emergency solution is always available. In general, it consists of an emergency call bell and/or a transparent alphabet board. Obviously, the more sophisticated the AAC system, the greater the need for specialized support. In our study, no patient or caregiver made a specific request for support after the short training period. The long daily usage observed in our study combined with the LeMMS’s easy application should indicate that little support is needed from highly specialized and trained personnel. In fact, it is worth noting that, before the study commenced, all PwALS were already using an AAC system, so the only novelty in this study was the new type of sensor we provided them.

### Impact on family caregiver burden

An important issue of the LeMMS is the easy positioning of the device for the caregivers. In fact, they need only to find the most appropriate finger position by moving the sensor, below it, until the proper leverage point, corresponding to the smallest activation force, is found.

It is well known that the involvement of the family in the palliative care process encourages family members to use AAC devices at home without this adding to their sense of burden [35]. Although in this study we did not address caregivers’ satisfaction via a specific questionnaire, the fact that no side effects or set-up/technical problems were reported during follow-up should imply that the caregivers had a positive perception of the new sensor. This positive perception could also be due to enhanced motivation as a result of their active involvement both in the training and follow-up.

### Single switch sensor versus high technology sensors

The experienced therapists involved in this study during their intervention explored different technological solutions to allow our patients to continue to communicate. ETCD and BCI technology prescription were two possible alternative options to the LeMMS. It is well known that an access system such as eye control is faster than scanning-access for PwALS. In particular, Gibbons et al. [26] reported that PwALS were about three times faster with eye tracking than single switch scanning in writing a text.

However, the cost disparity between switch- and eye-controlled technologies is an important variable in clinical decision-making. Actually, the cost of high technology devices is considered a material barrier to their application and diffusion [14, 35]. In Italy, the cost ratio between scanning and eye control technologies is about 1 to 15, although this is expected to decrease thanks to recent technological advances [9]. Further, due to the frequent discrepancy between the focus of attention and the user’s direction of gaze (the “Midas touch problem”), some authors reported that error rates for PwALS were higher for eye control than for single switch scan [26, 36]. The same authors found no difference in the perceived efficiency between patients using a switch sensor or ETCD. In addition, there can be problems of accuracy with some ETCD devices and their complex calibration and set-up can complicate the handling [37]. Apart from patient-related factors (e.g. oculomotor dysfunction) that can impede ETCD application, the sensitivity of the infrared sensitive camera of ETCD technology to ambient infrared light reduces the usefulness of these devices in an outdoor setting. This points to LeMMS as the preferable choice overall for those patients who could benefit from a more portable version of the AAC device. In practice, we recommended ETCD only for those patients in whom the LeMMS did not work for communication.

Finally, BCI could in theory be an alternative to ETCD in PwALS in whom the usability of the AAC system is compromised by oculomotor dysfunction, gaze fatigue or loss of eye movement control. However, the feasibility of BCI for patients with such a rapidly progressing disease is undermined by the very long training sessions required to reach an adequate level of accuracy [14]. In addition, a long time is needed to set up an EEG-BCI [38]. For these reasons we did not consider BCI as a practical choice.

## Limitations

Limitations of this study regard mainly the assessment protocol of patients. The study was not blinded because the therapists administering the assessment protocol were the same who applied the LeMMS and provided instructions on its use to the patients and caregivers. An additional limitation is that the scores regarding satisfaction and adverse events were reported by the main caregiver at the follow-up call. Hence, it is possible that this feedback may not fully reflect the true opinion of the patients. However, the administration of QUEST 2.0, GRC and collection of adverse events by phone call is common practice in studies with PwALS [33, 39, 40] mainly because, as the disease progresses, it is very difficult, if not impossible, for patients to attend the tertiary clinical care center, thus preventing them from enrolling in clinical trials or benefiting from specialized care and management [41].

## Conclusions

High technology AAC devices are not conceived to prolong the survival of PwALS but to enhance their quality of life and autonomy for the remaining lifetime. Despite barriers to the use of this technology, the new switch sensor we developed enabled PwALS to use their communication aids for a prolonged time even in the advanced phase of disease. The LeMMS is of easy application, reliable and cheap, thus representing an intermediate alternative to more sophisticated and costly devices. In addition, most patients reported high satisfaction with its usage. Thus, this device could be very useful in assisting and enhancing the communication between the patients and health professionals and caregivers (also as regards end of life decisions) thus improving the quality of life of patients and their families.

## Data Availability

The datasets used and analyzed during the current study are available from the corresponding author on reasonable request.

## Abbreviations

AAC: Augmentative Alternative Communication
ALSFRS-R: Amyotrophic Lateral Sclerosis Functional Rating Scale-Revised
AUC: Area under the curve
BCI: Brain-Computer Interface
ETCD: Eye Tracking Communication Device
GRC: Global Rating of Change scale
LeMMS: Lever Magnetic-spring Mechanical Switch
PwALS: People with Amyotrophic Lateral Sclerosis
QUEST 2.0: Quebec User Evaluation of Satisfaction with Assistive Technology questionnaire 2.0
ROC: Receiver operating characteristic
SD: Standard Deviation

## References

[1] Colombo R, Mazzini L, Mora G, Parenzan R, Creola G, Pirali I (2000). Measurement of isometric muscle strength: a reproducibility study of maximal voluntary contraction in normal subjects and amyotrophic lateral sclerosis patients. Med Eng Phys.

[2] Murphy J (2004). Communication strategies of people with ALS and their partners. Amyotroph Lateral Scler Other Motor Neuron Disord.

[3] Brownlee A, Palovcak M (2007). The role of augmentative communication devices in the medical management of ALS. NeuroRehabilitation.

[4] Ball LJ, Beukelman DR, Pattee GL (2004). Communication effectiveness of individuals with amyotrophic lateral sclerosis. J Commun Disord.

[5] Beukelman D, Fager S, Nordness A (2011). Communication support for people with ALS. Neurol Res Int.

[6] Beukelman DR, Fager S, Ball L, Dietz A (2007). AAC for adults with acquired neurological conditions: a review. Augment Altern Commun.

[7] Schlosser RW, Balandin S, Hemsley B, Iacono T, Probst P, von Tetzchner S (2014). Facilitated communication and authorship: a systematic review. Augment Altern Commun..

[8] Light J, McNaughton D (2014). From basic to applied research to improve outcomes for individuals who require augmentative and alternative communication: potential contributions of eye tracking research methods. Augment Altern Commun.

[9] Caligari M, Godi M, Guglielmetti S, Franchignoni F, Nardone A (2013). Eye tracking communication devices in amyotrophic lateral sclerosis: impact on disability and quality of life. Amyotroph Lateral Scler Frontotemporal Degener.

[10] McCane LM, Sellers EW, McFarland DJ, Mak JN, Carmack CS, Zeitlin D (2014). Brain-computer interface (BCI) evaluation in people with amyotrophic lateral sclerosis. Amyotroph Lateral Scler Frontotemporal Degener.

[11] Thompson DE, Blain-Moraes S, Huggins JE (2013). Performance assessment in brain-computer interface-based augmentative and alternative communication. Biomed Eng Online.

[12] Cipresso P, Carelli L, Solca F, Meazzi D, Meriggi P, Poletti B (2012). The use of P300-based BCIs in amyotrophic lateral sclerosis: from augmentative and alternative communication to cognitive assessment. Brain Behav.

[13] Anson D, Moist P, Przywara M, Wells H, Saylor H, Maxime H (2006). The effects of word completion and word prediction on typing rates using on-screen keyboards. Assist Technol.

[14] Linse K, Aust E, Joos M, Hermann A (2018). Communication matters-pitfalls and promise of Hightech communication devices in palliative Care of Severely Physically Disabled Patients with Amyotrophic Lateral Sclerosis. Front Neurol.

[15] Craig A, Tran Y, McIsaac P, Boord P (2005). The efficacy and benefits of environmental control systems for the severely disabled. Med Sci Monit.

[16] Alden DG, Daniels RW, Kanarick AF (1972). Keyboard Design and Operation: A Review of the Major Issues. Human Factors. J Hum Factors Ergon Soc.

[17] Nagurka ML, Marklin R (1999). Measurement of impedance characteristics of computer keyboard keys. Proceedings of the 7th IEEE Mediterranean Conference on Control and Automation (MED99), Haifa, Israel.

[18] Nagurka M, Marklin R (2005). Measurement of stiffness and damping characteristics of computer keyboard keys. J Dyn Syst Meas Control.

[19] Cedarbaum JM, Stambler N, Malta E, Fuller C, Hilt D, Thurmond B (1999). The ALSFRS-R: a revised ALS functional rating scale that incorporates assessments of respiratory function. BDNF ALS Study Group (Phase III). J Neurol Sci.

[20] Kendall FP (2006). I muscoli: funzioni e test con postura e dolore.

[21] Demers L, Weiss-Lambrou R, Ska B (2002). The Quebec User Evaluation of Satisfaction with Assistive Technology (QUEST 2.0): An overview and recent progress. Technol Disabil.

[22] Kamper SJ, Maher CG, Mackay G (2009). Global rating of change scales: a review of strengths and weaknesses and considerations for design. J Man Manip Ther.

[23] Akobeng AK (2007). Understanding diagnostic tests 3: receiver operating characteristic curves. Acta Paediatr.

[24] Froud R, Abel G (2014). Using ROC curves to choose minimally important change thresholds when sensitivity and specificity are valued equally: the forgotten lesson of Pythagoras. Theoretical considerations and an example application of change in health status. PLoS One.

[25] Gryfe P, Kurtz I, Gutmann M, Laiken G (1996). Freedom through a single switch: coping and communicating with artificial ventilation. J Neurol Sci.

[26] Gibbons C, Beneteau E (2010). Functional Performance Using Eye Control and Single Switch Scanning by People With ALS. Perspect Augment Altern Commun.

[27] Applebaum EV, Breton D, Feng ZW, Ta AT, Walsh K, Chassé K (2017). Modified 30-second Sit to Stand test predicts falls in a cohort of institutionalized older veterans. PLoS One.

[28] Darvishi S, Gharabaghi A, Ridding MC, Abbott D, Baumert M (2018). Reaction time predicts brain-computer Interface aptitude. IEEE J Transl Eng Health Med.

[29] Baxter S, Enderby P, Evans P, Judge S (2012). Barriers and facilitators to the use of high-technology augmentative and alternative communication devices: a systematic review and qualitative synthesis. Int J Lang Commun Disord.

[30] Moorcroft A, Scarinci N, Meyer C (2018). A systematic review of the barriers and facilitators to the provision and use of low-tech and unaided AAC systems for people with complex communication needs and their families. Disabil Rehabil Assist Technol.

[31] Bailey RL, Parette HP, Stoner JB, Angell ME, Carroll K (2006). Family members’ perceptions of augmentative and alternative communication device use. Lang Speech Hear Serv Sch.

[32] Cooper L, Balandin S, Trembath D (2009). The loneliness experiences of young adults with cerebral palsy who use alternative and augmentative communication. Augment Altern Commun.

[33] Spataro R, Ciriacono M, Manno C, La Bella V (2014). The eye-tracking computer device for communication in amyotrophic lateral sclerosis. Acta Neurol Scand.

[34] Dattilo J, Estrella G, Estrella LJ, Light J, McNaughton D, Seabury M (2008). «I have chosen to live life abundantly»: perceptions of leisure by adults who use augmentative and alternative communication. Augment Altern Commun..

[35] Romano N, Chun RYS (2018). Augmentative and Alternative Communication use: family and professionals’ perceptions of facilitators and barriers. Codas.

[36] Hansen JP, Tørning K, Johansen AS, Itoh K, Aoki H (2004). Gaze typing compared with input by head and hand. Proceedings of the 2004 symposium on Eye tracking research & applications.

[37] Ball LJ, Nordness AS, Fager SK, Kersch K, Mohr B, Pattee GL (2010). Eye gaze access of AAC technology for people with amyotrophic lateral sclerosis. Journal of medical speech-language pathology.

[38] Guy V, Soriani M-H, Bruno M, Papadopoulo T, Desnuelle C, Clerc M (2018). Brain computer interface with the P300 speller: usability for disabled people with amyotrophic lateral sclerosis. Ann Phys Rehabil Med.

[39] Vitacca M, Comini L, Assoni G, Fiorenza D, Gilè S, Bernocchi P (2012). Tele-assistance in patients with amyotrophic lateral sclerosis: long term activity and costs. Disabil Rehabil Assist Technol.

[40] Hobson EV, Baird WO, Cooper CL, Mawson S, Shaw PJ, Mcdermott CJ (2016). Using technology to improve access to specialist care in amyotrophic lateral sclerosis: a systematic review. Amyotroph Lateral Scler Frontotemporal Degener..

[41] Mannino M, Cellura E, Grimaldi G, Volanti P, Piccoli F, La Bella V (2007). Telephone follow-up for patients with amyotrophic lateral sclerosis. Eur J Neurol.

